# Research on enterprise business model and technology innovation based on artificial intelligence

**DOI:** 10.1186/s13638-021-02025-y

**Published:** 2021-07-03

**Authors:** Sunping Qu, Hongwei Shi, Huanhuan Zhao, Lin Yu, Yunbo Yu

**Affiliations:** 1 School of Management, JiangSu University, Zhenjiang, 212013 China; 2 College of Management, Wuxi Institute of Technology, Wuxi, 214121 China

**Keywords:** Technological innovation, Business model design, Business performance, Enterprise size, Artificial intelligence

## Abstract

Small- and medium-sized enterprises (SEMs) are the important part of economic society whose innovation activities are of great significance for building innovative country. In order to investigate how technological innovation (TI) and business model design (BMD) affect the business performance of SMEs, samples of 268 SMEs in the artificial intelligence industry and hierarchical regression models are used in the analysis. The results indicate that TI, BMD, and the matching of them have different effects on the innovation of SMEs of different sizes. These findings are helpful for enriching the theory of the fit between TI and BMD and providing theoretical guidance for the innovation activities in SEMs.

## Introduction

As COVID-19 sweeps through the world, the world economy is hit hard. But many companies in the field of artificial intelligence (AI) have achieved positive growth. In particular, many small- and medium-sized enterprises (SEMs) in the AI industry have grown rapidly. As an important part of economy, SEMs are numerous and provide a large number of jobs. They play an irreplaceable role in world economy. In order to survive and develop in the new crown epidemic, SEMs have a strong impetus for innovation. Innovation includes not only technological innovation (TI) but also business model design (BMD). TI, however, does not necessarily bring profits to enterprises, which also needs to combine with BMD. Chesbrough & Rosenbloom stated that TI can bring profits to enterprises due to BMD plays an important role [1]. BMD promotes the transformation and upgrading in China. Business model is an important approach of transforming TI into commercial value. Without business model transforming innovative technology into the products and services which customers really want, TI is just a waste of money, which cannot be a driving force for the healthy development of enterprises. Therefore, SMEs should proceed with TI and meanwhile implement the business model which is compatible with the TI.

TI and BMD are the hotspots of academic research. Scholars have put forward some meaningful research results, but the following research gaps still exist. First, from the perspective of research content, although the existing literature analyzed the effects of the fit between TI and BMD on enterprise performance, few studies take the types of TI and BMD into account. Second, from the perspective of research object, the existing literature studied the fit between TI and BMD in large enterprises, but paid little attention to SEMs. Existing research shows that there is a difference between the innovation of large enterprises and that of small and medium enterprises. Therefore, the academic community should research the innovation behaviors in SEMs [2]. Third, from the perspective of research methods, the main research method in existing literature is case study. Case study is good at discovering new theories, but whether the results of the case analysis are universal remains to be further tested [3]. However, the existing studies lack quantitative research, especially the research of the interaction effect of TI and BMD on BP in SEMs which is still at an infant stage [4]. To fill up these research gaps, this study collects data from 268 SEMs in the artificial intelligence industry in China with questionnaire survey, and uses hierarchical regression analysis to explore the effects of different types of TI and BMD and the fit between the two on BP in SEMs.

The key contributions of this work are:

1. By analyzing the existing literature, this work summarizes the impact of technological innovation, business model design, and the interaction between the two on enterprise innovation performance, and clarifies whether these conclusions are applicable to SMEs, which needs to be further tested.
2. This paper collects data with questionnaires, uses empirical analysis to study the impact of technological innovation, business model design, and the interaction of the two on the innovation performance of SMEs, and considers the impact of enterprise scale.
3. Based on the results of the empirical analysis, the article puts forward theoretical guidelines for the implementation of technological innovation and business model design activities for SMEs.

The remainder of the paper is organized as follows. In the next section, we review the literature on TI, BMD and BP that is relevant to our context, and develop our hypotheses. In Sect. 3, we describe our empirical approach and outline our sample of firms. In Sect. 4, this paper provides our results of hypotheses testing. In Sect. 5, we discuss the theoretical contributions and managerial implications and also provide promising areas for future research.

## Related work

Drawing on the research of Zott et al., this paper assumes that the trading environment contains three participants: core company, customer, supplier or partner. *F* refers to core company, which is the research object. *i* represents the i-th supplier or partner. The value of *i* ranges from 1 to *I*, which indicates that there is a total of *I* suppliers or partners. *t* represents the t-th transaction, which ranges from 1 to *T*. It means that there is a total of *T* types of transactions. *n(t)* represents the volume of the t-th transaction. The gains that the core company *F* receives from a particular transaction “*t*” is expressed by the following formula:

1 $${V_F}(t) = P(t) + \sum\limits_{i = 1}^I {{R_i}(t)} - \sum\limits_{i = 1}^I {{C_i}(t)} - {C_F}(t)$$

In which, *VF*(*t*) is the income that core company *F* gets from a single specific transaction *t*. *P*(*t*) is the fee paid by the customer to the core company *F* in the *t*th transaction. *Ri*(*t*) is the income that the core company *F* received from the *i*th supplier or partner in the *t*th transaction. $\sum\limits_{i = 1}^I {{R_i}(t)}$ is the sum of the income of core company *F* from all suppliers or partners in the *t*th transaction. *C*_*i*_(*t*) is the fee paid by the core company *F* to the *i*th supplier or partner in each transaction. $\sum\limits_{i = 1}^I {{C_i}(t)}$ is the fee paid by the core company *F* to suppliers or partners in the *t*th transaction. *CF*(*t*) is the cost of the core company in the *t*th transaction, including financial costs and intellectual costs [5].

The total value (*TV*) of the core company *F* in the business model is:

2 $$TV = \sum\limits_{t = 1}^T {[{V_F}(t) \times n(t)]}$$

In which, *n*(*t*) is the volume of the t-th transaction. The total income of the company is positively affected by product/service price (P(t)), company’s income from partners and suppliers (*Ri*(*t*)), quantity of transaction types (*T*), and volume of the *t*th transaction (*n*(*t*)), and is negatively affected by the cost consumed by partners and suppliers (*Ci*(*t*)), and the cost of core company (*CF* (*t*)) [6]. In the following part, this paper will analyze the effects of TI, BMD and the interaction between the two on business performance based on the framework proposed by Zott et al.

TI is the process which includes the generation of new ideas, R&D, trial production, manufacturing and commercialization. As mentioned above, TI can be classified into ITI and RTI [7]. ITI improves existing technologies and products to meet customer’s current needs, so as to improve business performance [8–10]. It reduces the cost of products and improves the quality of existing products by improving existing technology continuously [11]. When the cost of the product is reduced, on one hand, the enterprise would choose to keep price unchanged (*P*(*t*) →), and increase the profit of the enterprise (*VF*(*t*)↑) because of cost reduction (*CF*(*t*)↓) [12, 13]. On the other hand, it could also choose to reduce the price of the products (*P*(*t*)↓) to attract price-sensitive consumers, which will increase the purchase volume (*n*(*t*)↑), thus increasing the total income of the enterprise (*TV*↑) [14, 15]. When the quality of product/service is improved, the enterprise can increase the price of the product (*P*(*t*)↑) [16]. Reliable product quality will also bring good reputation to the enterprise, attract more consumers, and enlarge sales amount(*n*(*t*)↑) [17]. Therefore, excellent quality will increase the price and sales amount. In addition, in the supply chain, companies can enhance their bargaining power thanks to ITI, so that the company’s expenditure on suppliers or partners will reduce (*Ci*(*t*)↓), and the income of the focal firm will increase (*Ri*(*t*)↑). Thus, ITI has an obviously positive influence on enterprise performance due to *P*(*t*)↑, *Ci*(*t*)↓, *Ri*(*t*)↑, *CF*(*t*)↓ and *n*(*t*)↑ [18].

RTI targets new potential markets, which enable companies to enter new market successfully or to redefine the existing industries [19–21]. It provides new products for customers, increases the number of types of transactions (*T*↑) and provides for customers with unique products to increase the switching cost of customers, which creates a lock-in effect, so core companies can charge customers at higher price (*P*(*t*)↑) (at least initially). When it comes to suppliers or partners, core enterprises can first enter the market as a result of RTI, and gain the right to speak in the supply chain to have a high bargaining power [22, 23]. This enables the enterprise to increase income (*Ri*(*t*)↑) and reduce expenditure (*Ci*(*t*)↓) in the process of cooperation with suppliers and partners. As a breakthrough innovator, even a new market creator, enterprises may experience limited initial transactions (*n*(*t*)↓). As the markets have undergone earth-shaking changes nowadays, consumers become more willing to accept RTI [24–26]. Therefore, RTI has a significantly positive effect on enterprise performance because of *P*(*t*)↑, *Ri*(*t*)↑, *Ci*(*t*)↓, and *T*↑.

The scale of the enterprise plays a moderating role in the process of TI affecting enterprise performance. First, enterprises with different sizes have different innovation advantages [27]. Large-scale enterprises with a sound management system and good R&D capability can ensure that TI develops along the path of reducing costs and improving production processes [28]. Therefore, as the size of the enterprise increases, the ability to implement ITI is enhanced. While smaller enterprises have strong sense of innovation, flexible organizational structure, smooth internal communication, and rapid response to environmental changes. They often choose to entrust external organizations to develop or implement collaborative R&D for poor innovative resources and weak innovative capabilities. Collaborative innovation with external organizations enables enterprises to have access to a wealth of heterogeneous knowledge which facilitates ITI. Therefore, as the size of the enterprise becomes smaller, the ability to implement RTI is enhanced.

Moreover, both profitability of ITI and RTI are influenced by the size of the enterprises. Large-scale enterprises can expand the benefits of ITI due to scale effect. As mentioned earlier, ITI improves existing technologies, which can reduce the unit cost of products and services and improve the quality of them. In large-scale enterprises, when the sales volume is large (*n*(*t*)↑+), they can enjoy benefits of lower unit cost (*CF*(*t*)↓+), so enterprises can obtain more profit (*VF*(*t*)↑). Here, the "+" and "−" indicate the "positive" and "negative" moderating effect of enterprise scales. Large-scale enterprises have scale effect and can amplify the impact of price changes (*P*(*t*)↑+), which brings higher economic returns (*TV*↑) to enterprises owing to scale effect. In the face of suppliers or partners, large-scale enterprises have greater bargaining power and more speaking rights than small companies, so in the process of working with suppliers and partners, bigger companies can get more revenue (*Ri*(*t*)↑+) and pay less (*Ci*(*t*)↓+) to suppliers or partners. Because of scale effect, the R&D costs of unit products in large-scale enterprises are lower than SMEs (*CF*(*t*)↓+). Therefore, as the size of the enterprise becomes larger, the role of ITI in enterprise performance will become more important because of *P*(*t*)↑+, *Ri*(*t*)↑+, *Ci*(*t*)↓+, *CF*(*t*)↓+, *n*(*t*)↑+.

Smaller enterprises can hardly compete with large-scale enterprises in the mainstream market due to their limited market development capabilities. Small enterprises often choose niche markets to find something to grip. In order to meet the demands of fringe market customers, they need to adopt new RTI to provide new products and services. RTI takes advantage of the knowledge that is far from the company's existing knowledge base, which results in more uncertainty and higher risk. Senior enterprise leaders focus on the profitability of new technologies, while technology developers focus on the novelty of technology, which lead to poor communication. Smaller-scale enterprises have fewer management levels, smooth communication, and even the top leaders of the company are directly responsible for R&D, which can effectively reduce the risk of poor communication between technical personnel and senior management. In large-scale enterprises, there are many organizational levels, and the communication between the upper and lower levels is ineffective. This may lead to RTI, but it may be unprofitable. As the size of the enterprise becomes larger, the risk of RTI increases, which leads to a decline in corporate profit(*P*(*t*)↑−, *Ri*(*t*)↑−, *Ci*(*t*)↓−, *T*↑−). Therefore, the scale of the enterprise negatively regulates the role of RTI.

From the above, this paper proposes the following assumptions:

H1a ITI positively affects BP.

H1b RTIs positively affects BP.

H1c The scale of the enterprise positively regulates the influence of ITI on BP.

H1c The scale of the enterprise negatively regulates the influence of RTI on BP.

Business model describes how an organization is associated with external stakeholders and how to trade with them to create value for all stakeholders. From the perspective of value creating, BMD can be divided into two types: efficiency-centered business model design (EBMD) and novelty-centered business model design (NBMD). EBMD creates value by optimizing value chain to improve transaction efficiency and reduce transaction costs. It focuses on improving transaction efficiency, weakening the impact of uncertainties in the environment, and reducing transaction risk and communication and coordination costs for all parties (*CF*(*t*)↓, *Ci*(*t*)↓), which allows the participants involved in the transaction to obtain higher returns (TV↑). By reducing transaction costs, enterprises can attract more price-sensitive consumers at a lower price, which increases the number of existing customers (*n*(*t*)↑) and then improves enterprise performance. Therefore, EBMD positively affects enterprise performance because of *CF*(*t*)↓, *Ci*(*t*)↓, and *n*(*t*)↑.

NBMD creates new business models and user experiences, which inspires consumers’ willingness to pay higher price, such as Apple's APP Store. NBMD increases enterprise performance. There are three reasons for this: Firstly, NBMD gives people a refreshing feeling, improves the reputation of the enterprise, expands user markets (*n*(*t*)↑), and increases the pricing power (*P*(*t*)↑). Secondly, the focal firm has first-mover advantage, so the stakeholders can generate higher conversion costs which enhance the bargaining power of core enterprises(*Ri*(*t*)↑, *Ci*(*t*)↓). Thirdly, NBMD makes the focal firm reconnect with old and new trading partners in new ways, which will enlarge the sales volume of products or services (*n*(*t*)↑). Therefore, NBMD positively affects enterprise performance due to *P*(*t*)↑, *Ri*(*t*)↑, *Ci*(*t*)↓, and *n*(*t*)↑.

The effect of BMD on enterprise performance is moderated by enterprise scale. Large-scale enterprises have strong market capabilities, sound trading mechanisms, and strong bargaining power, which can gain more information on products and services to reduce information asymmetry with their own advantages. This provides necessary conditions for enterprises to successfully implement EBMD and is conducive for them to adopt EBMD. Therefore, the effect of EBMD is strengthened in large-scale enterprises (*CF*(*t*)↓+, *Ci*(*t*)↓+, and *n*(*t*)↑+).

SMEs have high innovation passion and flexible organizational structure, which facilitates smaller firms to trade with partners in new ways. For example, in the anti-virus software market, when Kingsoft and Kaspersky provided paid anti-virus software, Qihoo 360, a provider of Internet and mobile security products and services, provided anti-virus software for free, which made Qihoo 360 gain a large number of customers and grew rapidly. On the contrary, Because of organizational inertia, medium-sized enterprises have greater resistance to implementing novel business models compared with small and micro-enterprises. The effect of NBMD in medium-sized enterprises has been weakened(*P*(*t*)↑−, *Ri*(*t*)↑−, *Ci*(*t*)↓−, *n*(*t*)↑−).

In summary, this paper proposes the following assumptions:

H2a EBMD affects BP positively.

H2b NBMD affects BP positively.

H2c The scale of the enterprise moderates the influence of EBMD on BP positively.

H2d The scale of the enterprise moderates the influence of NBMD on BP negatively.

The premise that innovators can earn profit from TI is the successful commercialization of TI according to the innovation profit theory. TI and BMD strengthen each other, because they have the same goal. Both of them create value for customers. TI and business model in the business ecosystem is reflected in the interaction mechanism of “push–pull.” In addition, the combination of TI and BMD increases the difficulty of imitating and reduces the possibility of competitors’ imitation. Chesbrough et al. stated that business model plays an important role in the process of TI monetization, and business model is a bridge between technology and its economic value. Yao Mingming and other researchers find that the fit between BMD and TI strategy can significantly improve the performance of latecomer enterprises in China in the process of technology catch-up, Therefore, the interaction of TI and BMD is beneficial to improve enterprise performance.

(1) Interaction between ITI and BMD.

As mentioned above, ITI has a remarkably positive effect on business performance due to *P*(*t*)↑, *Ci*(*t*)↓, *Ri*(*t*)↑, *CF*(*t*)↓, and *n*(*t*)↑. It focuses on the customers’ needs of the existing markets and keeps improving the existing technologies, so we can say that ITI is a kind of gentle innovation, which would not have a devastating impact on existing technologies and markets. When ITI and BMD interact with each other, ITI plays a supporting role which would not change the effect of BMD’s leading role.

The efficient business model concentrates on reducing communication coordination costs for all parties of the transaction (*n*(*t*)↑) by reducing uncertainty in the transaction process and enhancing transaction efficiency, which will improve the performance of core enterprises. When ITI matches with EBMD, the purpose of both is to reduce costs and improve efficiency. They have the same logic and can enhance the promotion of enterprise performance. Therefore, ITI and EBMD positively affect business performance due to *P*(*t*)↑, *Ci*(*t*)↓, *Ri*(*t*)↑, *CF*(*t*)↓, and *n*(*t*)↑. NBMD re-connects old trading partners and new trading partners in new ways. When NBMD matches with ITI, NBMD plays a leading role, and the fit between the two positively affects the performance of the core enterprise due to *P*(*t*)↑, *Ri*(*t*)↑, *Ci*(*t*)↓, *n*(*t*)↑.

Enterprise scale moderates the interactive effects between ITI and BMD on innovation performance. With a sound management system and good R&D capabilities, large-scale enterprises are good at implementing ITI and get more benefits from ITI. In addition, large-scale enterprises have strong market capabilities, sound trading mechanisms, and strong bargaining power, which can reduce the information asymmetry in transaction process that is helpful to carry out EBMD. Therefore, as the size of the enterprise becomes larger, the interaction between ITI and EBMD will be enhanced (*P*(*t*)↑+, *Ci*(*t*)↓+, *Ri*(*t*)↑+, *CF*(*t*)↓+ and *n*(*t*)↑+). Larger-scale enterprises are not always good at NBMD due to lower innovation spirit and greater inertia. As the size of the enterprise becomes larger, the effect of ITI on the performance of SMEs increases, while the effect of NBMD decreases. Therefore, it is uncertain how the enterprise scale regulates the interaction between ITI and NBMD. This paper proposes the following hypotheses:

H3a The interaction between ITI and EBMD affects BP positively.

H3b The interaction between ITI and NBMD affects BP positively.

H3c The enterprise scale adjusts the interaction between ITI and EBMD positively.

H3d1 The enterprise scale moderates the interaction between ITI and NBMD positively.

H3d2 The enterprise scale moderates the interaction between ITI and NBMD negatively.

It should be noted that H3d1 and H3d2 are the hypotheses that cannot coexist. If neither H3d1 nor H3d2 is supported, it indicates that the size of the enterprise does not have a moderating effect on the interaction between ITI and NBMD.

(2) Interaction between RTI and BMD.

As mentioned above, RTI has a significantly positive effect on business performance as a result of *P*(*t*)↑, *Ri*(*t*)↑, *Ci*(*t*)↓, and T↑. RTI is aimed at new potential markets. RTI usually provides customers with novel products and services that need new technologies far from firms’ knowledge base. It has a greater impact on existing technologies and markets. Therefore, RTI plays an important role in the process of fitting RTI with BMD. EBMD lays emphasis on the efficiency, not the novelty of business model and is a gentle innovation. Therefore, in the combination of RTI and EBMD, RTI plays a leading role. The fit between RTI and EBMD affects business performance positively because of *P*(*t*)↑, *Ri*(*t*)↑, *Ci*(*t*)↓, and T↑.

Unlike EBMD, NBMD connects new partners or old partners in new ways, which is a kind of radical innovation. The combination of RTI and NBMD has greater uncertainty. There are two views in current literature. One view holds that NBMD increases the difficulty of commercialization of RTI. Too much novelty increases uncertainty and may lead to the loss of stakeholders and customers. But Zott and Amit thought that this view was not always correct, because NBMD may contain familiar design elements. The other view holds that the unique combination of RTI and NBMD gives enterprises unique advantages which will create value for them. As mentioned above, NBMD establish high switching costs for stakeholders(*P*(*t*)↑、*Ci*(*t*)↓、*Ri*(*t*)↑), which make it easier for stakeholder to accept RTI (*n*(*t*)↑), and increase transaction type (*T*↑). Rhoads K.'s research showed that enterprises might be limited by resources and capability in the process of RTI, but NBMD could weaken the potential limitations of resources and capacity. Therefore, RTI and NBMD will have a positive joint effect on business performance because *P*(*t*)↑, *Ri*(*t*)↑, *Ci*(*t*)↓, *n*(*t*)↑, *T*↑.

As the scale of enterprises increases, they are usually not good at RTI for lack of innovation spirit and technical rigidity. As mentioned previously, small companies are good at implementing NBMD. The scale of the enterprise, therefore, negatively regulates the interaction between RTI and EBMD (*P*(*t*)↑-, *Ri*(*t*)↑-, *Ci*(*t*)↓-, *n*(*t*)↑-, *T*↑-). Large-scale enterprises have strong market capability, sound trading mechanisms, strong bargaining power, and access to more information of products and services, and are experts in EBMD. When the scale of enterprises increases, the ability of RTI would decrease, and the innovation ability of NBMD will be enhanced. Thus, it is uncertain how enterprise size influences the interaction on business performance between RTI and EBMD.

Therefore, this paper proposes the following assumptions:

H4a The interaction between RTI and EBMD affects BP positively.

H4b The interaction between RTI and NBMD affects BP positively.

H4c1 The enterprise scale moderates the interaction between RTI and EBMD positively.

H4c2 The enterprise scale moderates the interaction between RTI and EBMD negatively.

H4d The enterprise scale moderates the interaction between RTI and NBMD negatively.

**Fig. 1:**
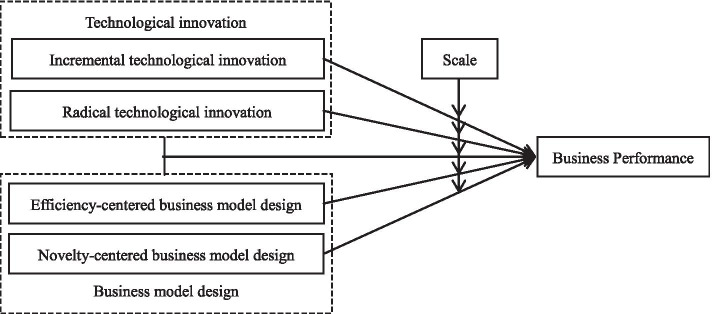
Theoretical hypothesis model

It should be noted that H4c1 and H4c2 are mutually exclusive. If neither H4c1 nor H4c2 is supported, it indicates that enterprise size does not moderate the interaction between RTI and EBMD. In summary, the role of innovation in business performance and the moderating effect of enterprise size are shown in Table 1 and Fig. 1. Table 1 illustrates the impact of technological innovation, business model design, and their interaction on the performance of SMEs. Figure 1 shows the theoretical hypothesis model of this article.

**Table 1:** Effects of innovation and enterprise size on trading factors

Innovation strategy	*P(t)*	*R*_*i*_*(t)*	*C*_*i*_*(t)*	*C*_*F*_*(t)*	*n(t)*	*T*
*ITI*	↑+	↑+	↓+	↓+	↑+	
*RTI*	↑−	↑−	↓−			↑−
*EBMD*			↓+	↓+	↑+	
*NBMD*	↑−	↑−	↓−		↑−	
*ITI* × *EBMD*	↑+	↑+	↓+	↓+	↑+	
*ITI* × *NBMD*	↑	↑	↓		↑	
*RTI* × *EBMD*	↑	↑	↓	↑		↑
*RTI* × *NBMD*	↑−	↑−	↓−		↑−	↑−

“↑” and “↓” indicate the elevated or reductive effect of innovation on trading factors, and “ + ” and “ − ” indicate the moderating effect of scale

## Methods

In order to test whether the above assumptions are correct, this paper constructs a regression model.

Direct effects model

3 $$\begin{aligned} B{P_{01}} & = {a_{01}} \times Y + {b_{01}} \times R + d{1_{01}} \times IC1 + d{2_{01}} \times IC2 + c{3_{01}} \times IC3 \\ & \quad + c{4_{01}} \times IC4 + c{5_{01}} \times IC5 + c{6_{01}} \times IC6 + c{7_{01}} \times IC7 \\ & \quad + {e_{01}} \times ITI + {f_{01}}\times RTI + {g_{01}}\times EBMD + {h_{01}}\times NBMD\;\;\; \\ \end{aligned}$$

*BP* is business performance of SMEs. *Y* is the age of enterprises*. R* is R&D intensity*. IC1, IC2, IC3, IC4, IC5, IC6* and *IC7* are dummy variables*,* which mean the types of AI industry. There are 8 types represented by 7 dummy variables. *ITI* is incremental technological innovation*. RTI* is radical technological innovation*. EBMD* is efficiency-centered business model design*. NBMD* is novelty-centered business model design*.* In order to measure the influence of the age of enterprise and investment in R&D on business performance, this paper takes the age of enterprise (*Y*), R&D intensity (*R*) and industry type (*IC*) as control variables.

Interaction model

4 $$\begin{aligned} B{P_{02}} & = {a_{02}} \times Y + {b_{02}} \times R + c{1_{02}} \times IC1 + c{2_{02}} \times IC2 \\ & \quad + c{3_{02}} \times CI3 + c{4_{02}} \times IC4 + c{5_{02}} \times IC5 + c{6_{02}} \times IC6 \\ & \quad + c{7_{02}} \times IC7 + {e_{02}} \times ITI + {f_{02}}\times RTI + {g_{02}}\times EBMD + {\text{ }}{h_{02}}\times NBMD \\ & \quad + {\text{ }}{j_{02}}\times ITI\times EBMD + {k_{02}}\times ITI\times NBMD + {\text{ }}{p_{02}}\times RTI\times EBMD{\text{ }} + {\text{ }}{q_{02}}\times RTI\times NBMD \\ \end{aligned}$$

In order to test the adjustment effect of enterprise scale, this article divides the whole sample into small and micro-enterprises and medium-sized enterprises. Compare the size of the standardized coefficients in the small and micro-enterprise model and the medium-sized enterprise model to illustrate the moderating effect of enterprise scale.

In small and micro-enterprises, the formulas for direct and indirect effects are as follows:

5 $$\begin{aligned} B{P_{11}} & = {a_{11}} \times Y + {b_{11}} \times R + d{1_{11}} \times IC1 + d{2_{11}} \times IC2 + c{3_{11}} \times IC3 \\ & \quad + c{4_{11}} \times IC4 + c{5_{11}} \times IC5 + c{6_{11}} \times IC6 + c{7_{11}} \times IC7 \\ & \quad + {e_{11}} \times ITI + {f_{11}}\times RTI + {g_{11}} \times EBMD + {h_{11}}\times NBMD \\ \end{aligned}$$

6 $$\begin{aligned} B{P_{12}} & = {a_{12}} \times Y + {b_{12}} \times R + c{1_{12}} \times IC1 + c{2_{12}} \times IC2 + c{3_{12}} \times CI3 \\ & \quad + c{4_{12}} \times IC4 + c{5_{12}} \times IC5 + c{6_{12}} \times IC6 + c{7_{12}} \times IC7 \\ & \quad + {e_{12}} \times ITI + {f_{12}}\times RTI + {g_{12}}\times EBMD + {\text{ }}{h_{12}}\times NBMD \\ & \quad + {\text{ }}{j_{12}}\times ITI\times EBMD + {k_{12}}\times ITI\times NBMD \\ & \quad + {\text{ }}{p_{12}}RTI\times EBMD\times {\text{ }} + {\text{ }}{q_{12}}\times RTI\times NBMD \\ \end{aligned}$$

In medium-sized enterprises, the formulas for direct and indirect effects are as follows:

7 $$\begin{aligned} B{P_{21}} & = {a_{21}} \times Y + {b_{21}} \times R + d{1_{21}} \times IC1 + d{2_{21}} \times IC2 + c{3_{21}} \times IC3 \\ & \quad + c{4_{21}} \times IC4 + c{5_{21}} \times IC5 + c{6_{21}} \times IC6 + c{7_{21}} \times IC7 \\ & \quad + {e_{21}} \times ITI + {f_{21}}\times RTI + {g_{21}}\times EBMD + {h_{21}}\times NBMD \\ \end{aligned}$$

8 $$\begin{aligned} B{P_{22}} & = {a_{22}} \times Y + {b_{22}} \times R + c{1_{22}} \times IC1 + c{2_{22}} \times IC2 + c{3_{22}} \times CI3 \\ & \quad + c{4_{22}} \times IC4 + c{5_{22}} \times IC5 + c{6_{22}} \times IC6 + c{7_{22}} \times IC7 \\ & \quad + {e_{22}} \times ITI + {f_{22}}\times RTI + {g_{22}}\times EBMD + {\text{ }}{h_{22}}\times NBMD \\ & \quad + {\text{ }}{j_{22}}\times ITI\times EBMD + {k_{22}}\times ITI\times NBMD \\ & \quad + {\text{ }}{p_{22}}\times RTI\times EBMD{\text{ }} + {\text{ }}{q_{22}}\times RTI\times NBMD \\ \end{aligned}$$

### Variable measurement

In order to ensure the reliability and validity of the questionnaire, this paper tries to use mature questionnaire and modify it according to the purpose of this study. *ITI* and *RTI* are both measured by four items. The measurement items for *ITI* are “① improving existing products and services [20]; ② enhancing existing production efficiency [21]; ③ adding extended services to existing customers [22]; ④adapting the types of existing products and services [23].” *RTI* are measured by “①developing new products and services [25]; ②developing new markets [26]; ③opening up new sales channels [27]; ④introducing new processes and technologies [28].” The authors used exploratory factor analysis to optimize the questionnaire, eliminating the items with poor consistency and selecting eight items to measure *EBMD* and *NBMD* [29]. The measurement items of *EBMD* are “①high transaction speed [30]; ②low inventory costs of suppliers and partners [31]; ③simplifying transaction process [32]; ④reducing error rate [33]; ⑤reducing partner cost [34]; ⑥easy access for consumers to gain enterprise information [35]; ⑦providing product and service information to participants [36]; ⑧making transactions faster and more efficient [37]." The measurement items of *NBMD* are ①providing the combination of new products, services and information [38]; ②attracting new consumers [39]; ③providing innovative consuming rewards [40]; ④attracting consumers in innovative ways [41]; ⑤communicating participants in a creative way [42]; ⑥leading role in *NBMD* [43]; ⑦continuously introducing innovation [44]; ⑧the innovative business model [45].” Business performance items include ①customer loyalty [46]; ②sales growth rate [47]; ③profit margin [48, 49]; ④rate of return on investment [50, 51].” The answer of questionnaire is designed to utilize five-point Likert Scale [52, 53].

### Sample survey

Using a cross-sectional survey method, the date was collected by sending questionnaires to SMEs in the artificial intelligence industry located in the Yangtze Delta Region in China. Using a stratified random sampling method, we selected firms according to the criteria: firms that conduct business model design and technological innovation at the same time. For each firm, we administered the questionnaire to senior managers. In order to improve the reliability and validity of the data,47 firms were selected for a pretest. Finally, a total of 400 questionnaires were distributed via field survey and email, of which 268 valid questionnaires remained, the effective response rate of 67%. The detail of the firms surveyed is shown in Table 2. According to *Small and Medium Sized Enterprise Standardization Regulations* issued by Chinese government, small and micro-enterprises usually have a turnover of less than 20 million while medium-sized enterprises more than 2001 million. The sample enterprises include 158 small and micro-enterprises and 110 medium-sized enterprises.

**Table 2:** Basic situation of the sample

Type		Frequency	Proportion (%)
Industry type	Smart furniture	18	6.72
	Smart driving	16	5.97
	Smart security	70	26.12
	Smart medical	26	9.70
	Smart manufacturing	31	11.57
	Smart financial services	59	22.01
	Smart logistics	39	14.55
	other	9	3.36
Business age	Within 3 years	47	17.54
	4–6 years	53	19.78
	7–9 years	54	20.15
	10–12 years	75	27.99
	13 years or more	39	14.55
Turnover	Less than 3 million	39	14.55
	3–20 million	119	44.40
	20–30 million	41	15.30
	30–40 million	24	8.96
	More than 40 million	45	16.79
R&D intensity	Lean back	15	5.60
	Middle and lower	46	17.16
	Middle	102	38.06
	Middle and upper	65	24.25
	Leading	40	14.93

### Common method bias

In order to avoid common method biases, the project team took a series of measures to try to control the possibility of common method bias in the process of questionnaire design and data collection. This paper uses the Harman single factor test to test the common method bias by observing the results of non-rotating factor analysis. The test results show that the contribution rate of the first factor extracted before the rotation is 22.032%, which is lower than the critical value of 40%, indicating that the common method deviation is not significant.

### Reliability and validity analysis

#### Reliability test

In this paper, the Cronbach α coefficient is used to analyze the reliability of the five main variables in the questionnaire. The results are shown in Table 3. It can be seen from the table that the Cronbach α coefficients of the five variables all meet the standard requirement of 0.7. This means that the variables show good consistency internally and thus pass the reliability test.

**Table 3:** Cronbach α coefficient, correlation coefficient and square root of AVE

Latent variable	Cronbach *α* coefficient	*ITI*	*RTI*	*EBMD*	*NBMD*	*BP*
*ITI*	0.879	0.805				
*RTI*	0.874	0.773	0.799			
*EBMD*	0.797	0.478	0.508	0.683		
*NBMD*	0.856	0.592	0.680	0.680	0.774	
*BP*	0.857	0.693	0.770	0.650	0.675	0.775

The diagonal number is the square root of AVE

#### Validity test

(1) Content validity.

The scale in this article is a mature research scale obtained by rigorous bilingual translation. The author draws on the opinions given by experts, so the questionnaire has good content validity.

(2) Convergence validity.

With the help of AMOS 20.0 software, this paper uses the structural equation model to do confirmatory factor analysis on the variables. The results are shown in Table 4. It can be seen from Table 4 that although the economic performance value of χ^2^/df and the RMSEA value of innovation strategy and economic performance are slightly higher than the standard, all three types of variables have good convergence validity in general.

**Table 4:** Fitting index statistics of the measurement model

Test indicator	*χ*^2^/*df*	GFI	NFI	IFI	CFI	RMSEA
Standard value	< 5	> 0.9	> 0.9	> 0.9	> 0.9	< 0.1
*TI*	3.826	0.937	0.945	0.959	0.959	0.103
*BMD*	2.087	0.912	0.917	0.955	0.955	0.064
*BP*	7.902	0.974	0.967	0.971	0.971	0.161

(3) Discriminant validity.

In this paper, the discriminant validity test is carried out by using the comparison between the square root of AVE and the correlation coefficient between the dimensions. As shown in Table 3, the square roots of AVE of the five variables are, respectively, 0.805, 0.799, 0.683, 0.774, and 0.775, which are higher than the correlation coefficient between any two variables. This shows that the five variables have good discriminant validity.

## Results and discussion

Based on the data collected by the questionnaire survey, with the help of SPSS, this paper uses the hierarchical regression method to analyze the effects of *TI*, *BMD* and their interaction on business performance. The result is shown in Table 5.

**Table 5:** Results of regression analysis

Variable		Full sample (268)		Small and micro-enterprises (158)		Medium-sized enterprises (110)	
		M01	M02	M11	M12	M21	M22
Control variable							
*Y*		−0.020	−0.008	0.019	0.017	−0.073	−0.044
*R*		0.157***	0.135***	0.150*	0.124**	0.144*	0.123*
*IC*	*IC1*	−0.017	−0.043	−0.055	−0.081	−0.014	−0.009
	*IC2*	0.059	0.014	0.130	0.060	−0.132*	−0.098*
	*IC3*	0.039	−0.017	0.132	0.043	−0.087	−0.073*
	*IC4*	0.051	0.007	−0.008	−0.061	0.063	0.059
	*IC5*	0.051	0.008	0.078	0.010	0.002	0.012
	*IC6*	0.113	0.044	0.151	0.049	0.103	0.083*
	*IC7*	0.097	0.040	0.136	0.054	0.000	0.011
Independent variable							
*ITI*		0.193***	0.070*	0.109	0.026	0.400***	0.174***
*RTI*		0.262***	0.103**	0.243**	0.075	0.202*	0.114*
*EBMD*		0.194**	0.055	0.207*	0.062	0.306**	0.081
*NBMD*		0.211**	0.083*	0.244**	0.104*	0.004	0.005
*Interaction*							
*ITI* × *EBMD*		0.125***		0.108***		0.159***	
*ITI* × *NBMD*		0.102***		0.093**		0.105***	
*RTI* × *EBMD*		0.117***		0.125***		0.109***	
*RTI* × *NBMD*		0.127***		0.145***		0.065*	
*F* value		33.354***	25.861***	16.935***	13.010***	19.476***	12.794***
Adj *R*^2^		0.612	0.613	0.569	0.565	0.670	0.648

The dependent variable is the business performance of SMEs and all coefficients are normalized coefficients. Significant levels: 0.001 (***), 0.01 (**), 0.05 (*)

In Full sample model, M01 shows the direct impact of control variables, TI and BMD on BP. The formula is as follows

9 $$\begin{aligned} B{P_{01}} & = - 0.02 \times Y + 0.157 \times R - 0.017 \times IC1 + 0.059 \times IC2 \\ & \quad + 0.039 \times CI3 + 0.051 \times IC4 + 0.051 \times IC5 \\ & \quad + 0.113 \times IC6 + 0.097 \times IC7 + {\text{ }}0.193 \times ITI \\ & \quad + 0.262\times RTI + {\text{ }}0.194\times EBMD + {\text{ }}0.211\times NBMD \\ \end{aligned}$$

M02 shows the impact of the interaction of TI and BMD on BP. The formula is as follows:

10 $$\begin{aligned} B{P_{02}} & = - 0.008 \times Y + 0.135 \times R - 0.043 \times IC1 + 0.014 \\ & \quad \times IC2 - 0.017 \times CI3 + 0.007 \times IC4 + 0.008 \times IC5 \\ & \quad + 0.044 \times IC6 + 0.04 \times IC7 + 0.070 \times ITI + 0.103\times RTI \\ & \quad + 0.055\times EBMD + 0.083\times NBMD + 0.125\times ITIEBMD \\ & \quad + 0.102\times ITI\times NBMD + 0.117\times RTI\times EBMD + 0.127\times RTI\times NBMD \\ \end{aligned}$$

**Fig. 2:**
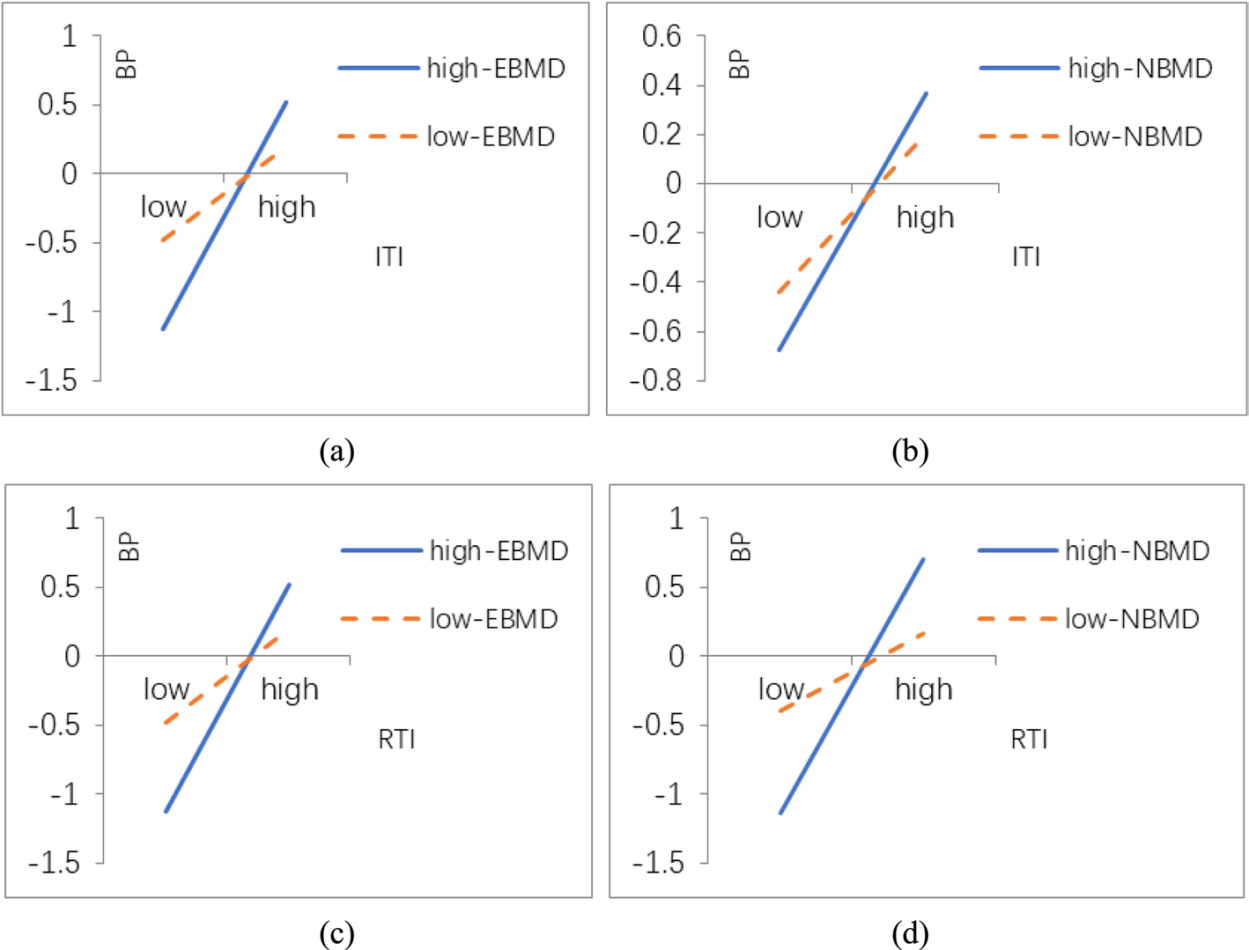
Business model design regulates the impact of technological innovation on corporate performance

It can be seen from the model M01 in Table 5 that the coefficients of *ITI*, *RTI*, *EBMD* and *NBMD* are all significant at the level of 0.01. It means that both *TI* and *BMD* can improve the performance to SMEs. This conclusion is consistent with the research conclusions of Li Yi, Li Jianli, Zhang Wei and others. Both *EBMD* and *NBMD* are beneficial to business performance. This conclusion is also consistent with the research conclusions of Yao Mingming, Li Wei and others that *BMD* positively affects business performance. Therefore, H1a, H1b, H2a, and H2b are confirmed. In model M02, the coefficients of *ITI* × *EBMD、ITI* × *NBMD、RTI* × *EBMD* and *RTI* × *NBMD* all are all significant at the level of 0.001. As shown in Fig. 2, the interaction between *TI* and *BMD* can bring great performance to enterprises for the full sample. The research conclusions of K. Rhoads and others also show that *RTI* and *NBMD* are more conducive to improve business performance [18]. Therefore, H3a, H3b, H4a, and H4b are confirmed. Existing researches reveal that the fit between *TI* and *BMD* is helpful to improve business performance, but there is no comparison effect between different types of *TI* and *BMD*. It is confirmed that both the fit between *ITI* and *EBMD* and the fit between *RTI* and *NBMD* can bring greater economic benefits to enterprises in model M02.

In Table 5, M11 demonstrated the direct impact of controlled variables, *TI* and *BMD* on the *BP* of small and micro-enterprises. The formula is as follows:

$$\begin{aligned} B{P_{11}} & = 0.019 \times Y + 0.15 \times R - 0.055 \times IC1 + 0.130 \times IC2 \\ & \quad + 0.132 \times CI3 - 0.008 \times IC4 + 0.078 \times IC5 \\ & \quad + 0.151 \times IC6 + 0.136 \times IC7 + {\text{ }}0.109 \times ITI \\ & \quad + 0.243\times RTI + 0.207\times EBMD + {\text{ }}0.244\times NBMD \\ \end{aligned}$$

M12 demonstrated the interaction of TI and BMD on the BP of small and micro-enterprises. The formula is as follows:

11 $$\begin{aligned} B{P_{12}} & = 0.017 \times Y + 0.124 \times R - 0.081 \times IC1 + 0.06 \times IC2 \\ & \quad + 0.043 \times CI3 - 0.061 \times IC4 + 0.01 \times IC5 + 0.049 \times IC6 \\ & \quad + 0.054 \times IC7 + {\text{ }}0.026 \times ITI + 0.075\times RTI + 0.062\times EBMD \\ & \quad + 0.104NBMD + 0.108\times ITI\times EBMD + 0.093\times ITI\times NBMD \\ & \quad + 0.125\times RTI\times EBMD + 0.145\times RTI\times NBMD \\ \end{aligned}$$

M21 shows the direct impact of control variables, *TI* and *BMD* on the *BP* of medium-sized enterprises. The formula is as follows:

12 $$\begin{aligned} B{P_{21}} & = - 0.073 \times Y + 0.144 \times R - 0.014 \times IC1 - 0.132 \\ & \quad \times IC2 - 0.087 \times CI3 + 0.063 \times IC4 + 0.002 \times IC5 \\ & \quad + 0.103 \times IC6 + 0 \times IC7 + {\text{ }}0.4 \times ITI \\ & \quad + 0.202\times RTI + {\text{ }}0.306\times EBMD + 0.004\times NBMD \\ \end{aligned}$$

M22 shows the impact of the interaction of *TI* and *BMD* on the *BP* of medium-sized companies. The formula is as follows:

13 $$\begin{aligned} B{P_{22}} & = - 0.044 \times Y + 0.123 \times R - 0.009 \times IC1 - 0.098 \times IC2 \\ & \quad - 0.073 \times CI3 + 0.059 \times IC4 + 0.012 \times IC5 + 0.083 \\ & \quad \times IC6 + 0.011 \times IC7 + 0.174 \times ITI + 0.114\times RTI \\ & \quad + 0.081\times EBMD + 0.005\times NBMD + 0.159\times ITI\times EBMD \\ & \quad + 0.105\times ITI\times NBMD + 0.109\times RTI\times EBMD + 0.065\times RTI\times NBMD \\ \end{aligned}$$

Comparing the coefficients of *ITI* in model M11 and M21, the coefficient of *ITI* in medium-sized enterprise is higher than the coefficient of *ITI* in small and micro-enterprise which is not significant at the level of 0.5. This means that as the size of the enterprise becomes larger, the role of *ITI* in business performance is enhanced. The scale of the enterprise moderates the effect of *ITI* in business performance positively, so H1c is supported. Similarly, the scale of the enterprise moderates the effect of *EBMD* on business performance positively, thus H2c is supported. In the M11 and M21 models, the coefficient of *RTI* in small and micro-enterprises is higher than that in medium-sized enterprises, which indicates that as the size of enterprises becomes larger, the effect of *RTI* on business performance will decrease. The scale of the enterprise moderates the effect of *RTI* on business performance negatively, therefore H1d is supported. Similarly, the scale of the enterprise moderates the effect of *NBMD* on business performance negatively, so H2d is supported. Existing researches show that both *ITI* and *RTI* have a promoting effect on business performance, but few of them take the size of the business into account. This study analyzes the moderation of enterprise size which expend the research on the effects of *TI* on business performance.

TI does not necessarily bring economic benefits to enterprises unless it is matched with *BMD*. It shows that different types of *TI* and *BMD* have different effects on business performance. The effects of the fit between TI and *BMD* on business performance vary as the size of enterprise changes. However, the existing literature rarely analyzes the moderation of enterprise size in the fit effect of *TI* and *BMD*. This paper studies the moderation of enterprise size based on the combination of *TI* and *BMD*. In model M12 and M22, the coefficient of *ITI* × *EBMD* in medium-sized enterprises is higher than that in small and micro-enterprises, which indicates that as the size of enterprises becomes larger, the interaction between *ITI* and *EBMD* will be enhanced. The scale of enterprises moderates the interaction between *ITI* and *NBMD* positively, so the results support H3c. Similarly, the scale of enterprises moderates the interaction between *ITI* and *NBMD* positively. H3d1 is supported while H3d2 is not supported. Comparing the coefficients of *RTI* × *EBMD* between M12 and M22, it can be seen that the coefficient in small and micro-enterprises is higher than that in medium-sized enterprises. It is clearly evident from Fig. 2 that as the size of enterprises becomes larger, the interaction between *RTI* and *EBMD* recedes. The size of enterprises moderates the interaction between *RTI* and *EBMD* negatively. Therefore, H4c2 is supported and H4c1 is not supported. Similarly, the scale of enterprises negatively regulates the interaction between *RTI* and *NBMD*, so H4d is supported.

## Conclusion

Innovation plays a vital role in the survival and development of SEMs. The role of TI, BMD, and the fit between the two in business performance has been widely recognized by the academic community. Previous researches studied the effects of TI and BMD in promoting business performance without taking enterprise size into account. They examined that the fit between TI and BMD can better promote the development of enterprises. However, few of them compared the fit effects of different types of TI and BMD, and even fewer investigated the change in fit effects of enterprises with different scales. Therefore, this paper collects data of 268 SEMs with questionnaires and uses hierarchical regression analysis to explore the effects of TI, BMD and the fit between the two on business performance. The research results show that different types of TI and BMD and the fit between the two have different effects on business performance of SEMs with different scales, which provides theoretical guidance for SMEs to carry out TI and BMD. The conclusions and implications display as follow:

The direct effects of different TI and BMD on business performance are positive in the whole sample. This suggests that for most enterprises, TI and BMD can bring good performance to them. However, when considering the size of the enterprise, the effect of TI and BMD on business performance is not always significant. In small and micro-enterprises, RTI and NBMD have a greater direct effect on business performance than ITI and EBMD. In medium-sized companies, the opposite is true. Therefore, SMEs should take enterprise size into account when formulating innovation strategy. Small and micro-enterprises should reinforce the novelty of TI and BMD, while medium-sized enterprises should focus on efficiency. In the view of interaction of innovation, in small and micro-enterprises, the coefficient of *RTI* × *BMD* is higher than that of the whole sample. Moreover, the coefficient of *RTI* × *NBMD* is the highest one. In contrast, in medium-sized companies, the coefficient of interaction between ITI and BMD is higher than that of the full sample. Furthermore, the coefficient of interaction between ITI and EBMD has the highest value. Therefore, enterprises need to take into account the fit between TI and BMD when implementing innovation strategies. And the effects of innovation strategies are moderated by enterprise sizes. Small and micro-enterprises need to pay attention to the fit between RTI and NBMD, while medium-sized enterprises should attach importance to the fit between ITI and EBMD. NBMD reduces the harm caused by RTI and the fit between the two is beneficial to improve the innovation performance of enterprises. This conclusion is supported in small and micro-enterprises, but is not supported in medium-sized enterprises. In small and micro-enterprises, the interaction between RTI and NBMD has the greatest effect on business performance, which shows that the fit between the two can better improves business performance. In medium-sized enterprises, the coefficient of *RTI* × *NBMD* is not significant at the level of 0.01. In brief, it depends on the size of the company whether the combination of RTI and NBMD improve business performance. The study enriches the theory of the fit between TI and BMD.

## Data Availability

Data sharing is not applicable to this article as no datasets were generated or analyzed during the current study.

## Abbreviations

SEMs: Small- and medium-sized enterprises
TI: Technological innovation
ITI: Incremental technological innovation
RTI: Radical technological innovation
BMD: Business model design
EBMD: Efficiency-centered business model design
NBMD: Novelty-centered business model design
BP: Business performance

## References

[1] Chesbrough H, Rosenbloom RS (2002). The role of the business model in capturing value from innovation: evidence from Xerox Corporation's technology spin-off companies. Ind. Corp. Change..

[2] Gatautis R, Vaiciukynaite E, Tarute A (2019). Impact of business model innovations on SME's innovativeness and performance. Balt. J. Manag..

[3] Guo H, Tang J, Su Z, Katz JA (2017). Opportunity recognition and SME performance: The mediating effect of business model innovation. R&D Manag..

[4] Sapra H, Subramanian A, Subramanian KV (2014). Corporate governance and innovation: theory and evidence. J. Financ. Quant. Anal..

[5] Baden-Fuller C, Morgan MS (2010). Business models as models. Long Range Plan..

[6] Rayna T, Striukova L (2016). From rapid prototyping to home fabrication: how 3D printing is changing business model innovation. Technol. Forecast. Soc. Chang..

[7] Zott C, Amit R, Massa L (2011). The business model: recent developments and future research. J. Manag..

[8] Taran Y, Boer H, Lindgren P (2015). A business model innovation typology. Decis. Sci..

[9] Wirtz BW, Pistoia A, Ullrich S, Göttel V (2016). Business models: origin, development and future research perspectives. Long Range Plan..

[10] Jiang C, Li R, Chen T, Xu C, Li L, Li S (2020). A two-lane mixed traffic flow model with drivers' intention to change lane based on cellular automata. Int. J. Bio-Inspired Comput..

[11] Brettel M, Strese S, Flatten TC (2012). Improving the performance of business models with relationship marketing efforts - An entrepreneurial perspective. Eur. Manag. J..

[12] Saebi T, Foss NJ (2015). Business models for open innovation: Matching heterogeneous open innovation strategies with business model dimensions. Eur. Manag. J..

[13] Zott C, Amit R (2007). Business model design and the performance of entrepreneurial firms. Organ. Sci..

[14] Mitchell D, Coles C (2003). The ultimate competitive advantage of continuing business model innovation. J. Bus. Strateg..

[15] Demil B, Lecocq X (2010). Business model evolution: in search of dynamic consistency. Long Range Plan..

[16] Zott C, Amit R (2008). The fit between product market strategy and business model: implications for firm performance. Strateg. Manag. J..

[17] Ferreras-Méndez JL, Newell S, Fernández-Mesa A, Alegre J (2015). Depth and breadth of external knowledge search and performance: the mediating role of absorptive capacity. Ind. Mark. Manage..

[18] Liu Z, Lang L, Hu B, Shi L, Huang B, Zhao Y (2021). Emission reduction decision of agricultural supply chain considering carbon tax and investment cooperation. J. Clean. Prod..

[19] He Z, Wong P (2004). Exploration vs. Exploitation: an empirical test of the ambidexterity hypothesis. Org. Sci..

[20] Zhu J, Shi Q, Wu P, Sheng Z, Wang X (2018). Complexity analysis of prefabrication contractors’ dynamic price competition in mega projects with different competition strategies. Complexity.

[21] Sun J, Lv X (2021). Feeling dark, seeing dark: mind–body in dark tourism. Ann. Tourism Res..

[22] Lv X, Liu Y, Luo J, Liu Y, Li C (2021). Does a cute artificial intelligence assistant soften the blow? The impact of cuteness on customer tolerance of assistant service failure. Ann. Tourism Res..

[23] Hu X, Chong H, Wang X (2019). Sustainability perceptions of off-site manufacturing stakeholders in Australia. J. Clean. Prod..

[24] Lv X, Li N, Xu X, Yang Y (2020). Understanding the emergence and development of online travel agencies: a dynamic evaluation and simulation approach. Internet Res..

[25] Liu S, Chan FTS, Ran W (2016). Decision making for the selection of cloud vendor: An improved approach under group decision-making with integrated weights and objective/subjective attributes. Expert Syst. Appl..

[26] Ran W, Liu S, Zhang Z (2020). A polling-based dynamic order-picking system considering priority orders. Complexity.

[27] Zhu J, Wang X, Wang P, Wu Z, Kim MJ (2019). Integration of BIM and GIS: Geometry from IFC to shapefile using open-source technology. Autom. Constr..

[28] Yang J, Li S, Wang Z, Dong H, Wang J, Tang S (2020). Using deep learning to detect defects in manufacturing: a comprehensive survey and current challenges. Materials..

[29] Sun J, Wang X, Xiong N, Shao J (2018). Learning sparse representation with variational auto-encoder for anomaly detection. IEEE Access..

[30] Yi B, Shen X, Liu H, Zhang Z, Zhang W, Liu S, Xiong N (2019). Deep matrix factorization with implicit feedback embedding for recommendation system. IEEE Trans. Ind. Inf..

[31] Lin B, Zhu F, Zhang J, Chen J, Chen X, Xiong N, Mauri JL (2019). A time-driven data placement strategy for a scientific workflow combining edge computing and cloud computing. IEEE Trans. Ind. Inf..

[32] Li H, Liu J, Liu RW, Xiong N, Wu K, Kim T (2017). A dimensionality reduction-based multi-step clustering method for robust vessel trajectory analysis. Sensors.

[33] Fang W, Yao X, Zhao X, Yin J, Xiong N (2016). A stochastic control approach to maximize profit on service provisioning for mobile cloudlet platforms. IEEE Trans Syst, Man Cybern. Syst..

[34] Chen Y, Zheng W, Li W, Huang Y (2021). Large group activity security risk assessment and risk early warning based on random forest algorithm. Pattern Recogn. Lett..

[35] Zhou Y, Tian L, Zhu C, Jin X, Sun Y (2020). Video coding optimization for virtual reality 360-degree source. IEEE J. Select. Top. Signal Process..

[36] Zhao J, Liu J, Jiang J, Gao F (2020). Efficient deployment with geometric analysis for mm wave UAV communications. IEEE Wireless Commun. Lett..

[37] Xiong Z, Xiao N, Xu F, Zhang X, Xu Q, Zhang K, Ye C (2020). An equivalent exchange based data forwarding incentive scheme for socially aware networks. J. Signal Process. Syst..

[38] Jiang Q, Shao F, Lin W, Gu K, Jiang G, Sun H (2018). Optimizing multistage discriminative dictionaries for blind image quality assessment. IEEE Trans. Multim..

[39] Li B, Liu Y, Zhang A, Wang W, Wan S (2020). A survey on blocking technology of entity resolution. J. Comput. Sci. Technol..

[40] Hu J, Zhang H, Liu L, Zhu X, Zhao C, Pan Q (2020). Convergent multiagent formation control with collision avoidance. IEEE Trans. Rob..

[41] Hu J, Zhang H, Li Z, Zhao C, Xu Z, Pan Q (2020). Object traversing by monocular UAV in outdoor environment. Asian Journal of Control..

[42] Hu J, Zheng B, Wang C, Zhao C, Hou X, Pan Q, Xu Z (2020). A survey on multi-sensor fusion based obstacle detection for intelligent ground vehicles in off-road environments. Front. Inform. Technol. Electron. Eng..

[43] Hu J, Wang M, Zhao C, Pan Q, Du C (2020). Formation control and collision avoidance for multi-UAV systems based on Voronoi partition. Sci. China Technol. Sci..

[44] Niu Z, Zhang B, Wang J, Liu K, Chen Z, Yang K, Zhou Z, Fan Y, Zhang Y, Ji D, Feng Y, Liu Y (2020). The research on 220GHz multicarrier high-speed communication system. China Commun..

[45] Zhang B, Niu Z, Wang J, Ji D, Zhou T, Liu Y, Feng Y, Hu Y, Zhang J, Fan Y (2020). Four-hundred gigahertz broadband multi-branch waveguide coupler. IET Microwaves Antennas Propag..

[46] Zhang B, Ji D, Fang D, Liang S, Fan Y, Chen X (2019). A novel 220-GHz GaN diode on-chip tripler with high driven power. IEEE Electron Device Lett..

[47] Liu Y, Zhang B, Feng Y, Lv X, Ji D, Niu Z, Yang Y, Zhao X, Fan Y (2020). Development of 340-GHz transceiver front end based on GaAs monolithic integration technology for THz active imaging array. Appl. Sci..

[48] Ma H, Yang G (2016). Adaptive Fault tolerant control of cooperative heterogeneous systems with actuator faults and unreliable interconnections. IEEE Trans. Autom. Control.

[49] Ma H, Xu L (2020). Decentralized adaptive fault-tolerant control for a class of strong interconnected nonlinear systems via graph theory. IEEE Trans. Autom. Control.

[50] Ma J, Xu LX, Yang GH (2021). Multiple environment integral reinforcement learning-based fault-tolerant control for affine nonlinear systems. IEEE Trans. Cybern..

[51] Zhang X, Jing R, Li Z, Li Z, Chen X, Su CY (2020). Adaptive pseudo inverse control for a class of nonlinear asymmetric and saturated nonlinear hysteretic systems. IEEE/CAA J. Automatica Sinica.

[52] Li A, Spano D, Krivochiza J, Domouchtsidis S, Tsinos CG, Masouros C, Chatzinotas S, Li Y, Vucetic B, Ottersten B (2020). A tutorial on interference exploitation via symbol-level precoding: overview, state-of-the-art and future directions. IEEE Commun. Surv. Tutorials..

[53] Wang S, Wang X, Meng F, Yang R, Zhao Y (2020). Investor behaviour monitoring based on deep learning. Behav. Inform. Technol..

